# Crystal structure of (5*Z*)-5-(2-hy­droxy­benzyl­idene)-1,3-thia­zolidine-2,4-dione

**DOI:** 10.1107/S2056989015021908

**Published:** 2015-11-21

**Authors:** Joel T. Mague, Shaaban K. Mohamed, Mehmet Akkurt, Sabry H. H. Younes, Mustafa R. Albayati

**Affiliations:** a Department of Chemistry, Tulane University, New Orleans, LA 70118, USA; b Chemistry and Environmental Division, Manchester Metropolitan University, Manchester M1 5GD, England; c Chemistry Department, Faculty of Science, Minia University, 61519 El-Minia, Egypt; d Department of Physics, Faculty of Sciences, Erciyes University, 38039 Kayseri, Turkey; e Department of Chemistry, Faculty of Science, Sohag University, 82524 Sohag, Egypt; f Kirkuk University, College of Science, Department of Chemistry, Kirkuk, Iraq

**Keywords:** crystal structure, thia­zolidinones, hydrogen bonding

## Abstract

The title compound, C_10_H_7_NO_3_S, crystallizes with four independent mol­ecules in the asymmetric unit with slightly different conformations; the dihedral angles between the six- and five-membered rings are 2.6 (1), 1.09 (9), 8.6 (1) and 6.2 (1)°. In the crystal, mol­ecules are linked by O—H⋯O and N—H⋯O hydrogen bonds, forming sheets lying parallel to (101).

## Related literature   

For synthesis and biological activities of thia­zolidinones, see: Singh *et al.* (1981 ▸); Bondock *et al.* (2007 ▸); Vicini *et al.* (2008 ▸); Behbehani & Ibrahim (2012 ▸).
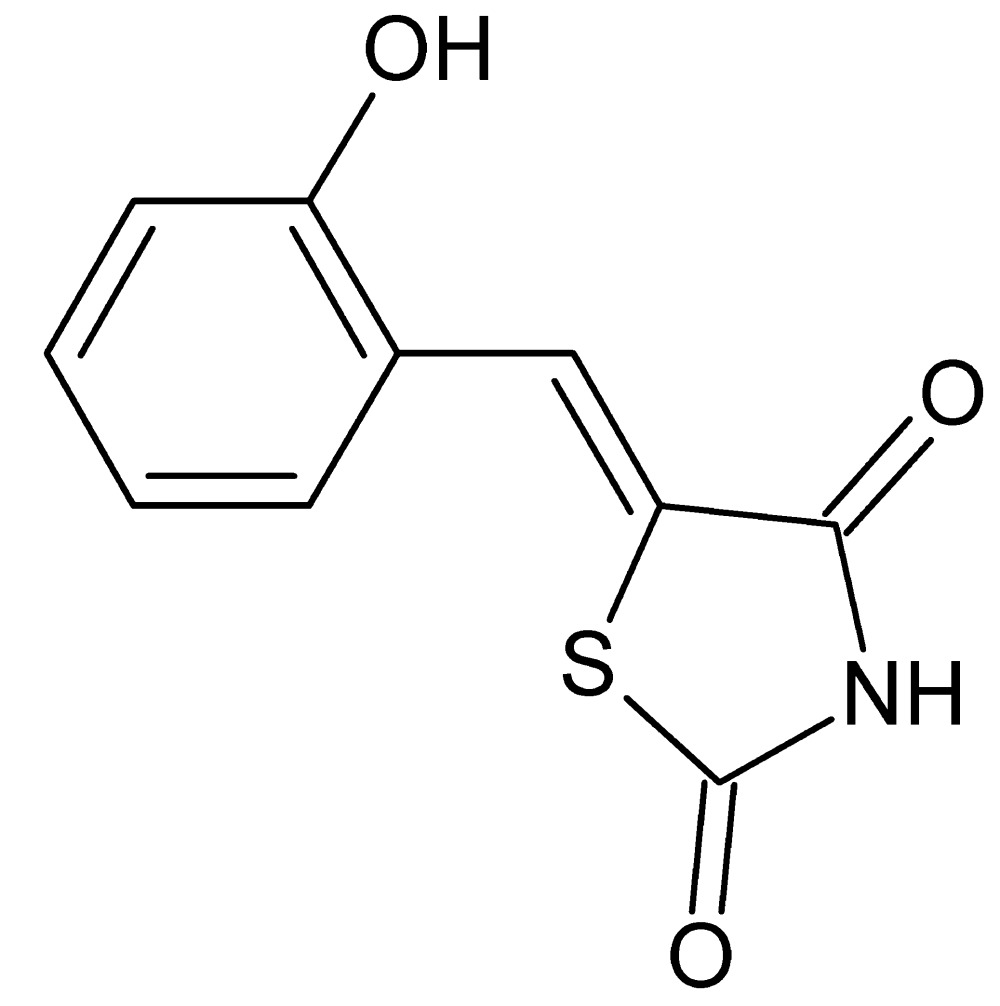


## Experimental   

### Crystal data  

C_10_H_7_NO_3_S
*M* *_r_* = 221.23
Triclinic, 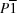

*a* = 7.2040 (7) Å
*b* = 13.6544 (14) Å
*c* = 18.9346 (18) Å
α = 90.226 (2)°
β = 95.531 (2)°
γ = 91.330 (2)°
*V* = 1853.3 (3) Å^3^
*Z* = 8
Mo *K*α radiation
μ = 0.33 mm^−1^
*T* = 150 K
0.22 × 0.11 × 0.08 mm

### Data collection  

Bruker SMART APEX CCD diffractometer
Absorption correction: multi-scan (*TWINABS*; Sheldrick, 2009 ▸) *T*_min_ = 0.93, *T*_max_ = 0.97
64240 measured reflections
9869 independent reflections
5466 reflections with *I* > 2σ(*I*)
*R*_int_ = 0.074

### Refinement  

*R*[*F* ^2^ > 2σ(*F* ^2^)] = 0.051
*wR*(*F* ^2^) = 0.125
*S* = 0.93
9869 reflections
541 parameters
H-atom parameters constrained
Δρ_max_ = 0.53 e Å^−3^
Δρ_min_ = −0.38 e Å^−3^

### 

Data collection: *APEX2* (Bruker, 2015 ▸); cell refinement: *SAINT* (Bruker, 2015 ▸); data reduction: *SAINT*; program(s) used to solve structure: *SHELXT* (Sheldrick, 2015*a* ▸); program(s) used to refine structure: *SHELXL2014* (Sheldrick, 2015*b* ▸); molecular graphics: *DIAMOND* (Brandenburg & Putz, 2012 ▸); software used to prepare material for publication: *SHELXL2014*.

## Supplementary Material

Crystal structure: contains datablock(s) global, I. DOI: 10.1107/S2056989015021908/is5432sup1.cif

Structure factors: contains datablock(s) I. DOI: 10.1107/S2056989015021908/is5432Isup2.hkl

Supporting information file. DOI: 10.1107/S2056989015021908/is5432Isup3.cml

**Fig. 1.**
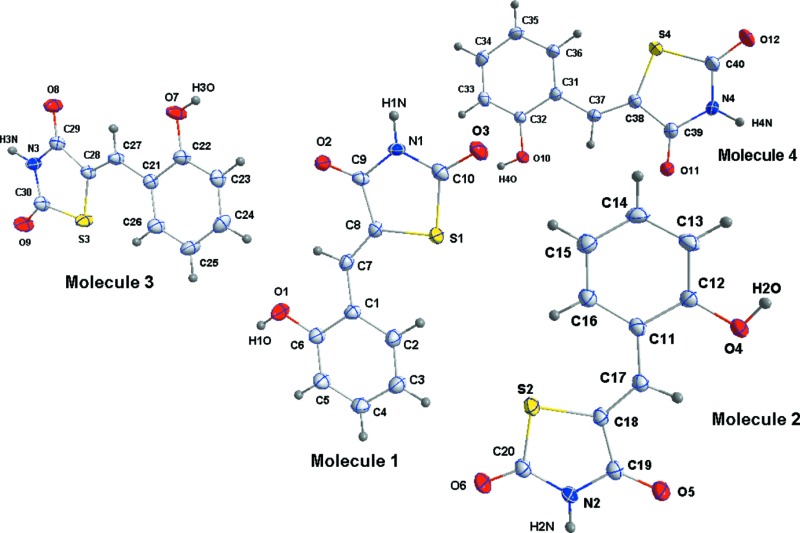
The asymmetric unit showing labeling scheme and 50% probability ellipsoids.

**Fig. 2.**
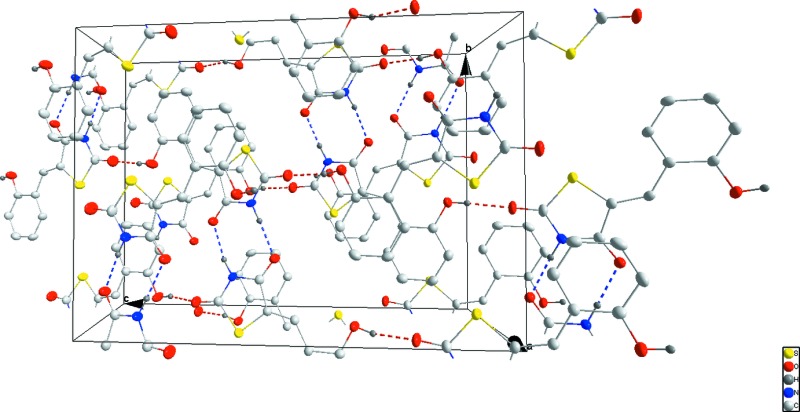
A packing diagram viewed down the a axis with O—H···O and N—H···N hydrogen bonds shown, respectively, as red and blue dotted lines.

**Fig. 3.**
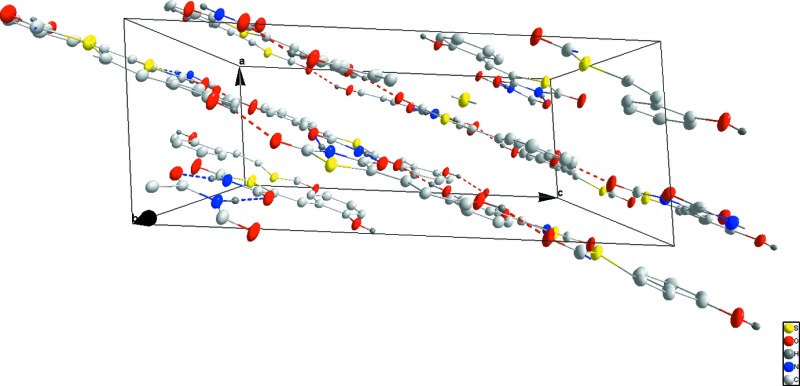
A packing diagram viewed down the b axis showing the sheet structure.

. DOI: 10.1107/S2056989015021908/is5432fig1.tif
The asymmetric unit showing labeling scheme and 50% probability ellipsoids.

a . DOI: 10.1107/S2056989015021908/is5432fig2.tif
A packing diagram viewed down the *a* axis with O—H⋯O and N—H⋯N hydrogen bonds shown, respectively, as red and blue dotted lines.

b . DOI: 10.1107/S2056989015021908/is5432fig3.tif
A packing diagram viewed down the *b* axis showing the sheet structure.

CCDC reference: 1437385

Additional supporting information:  crystallographic information; 3D view; checkCIF report

## Figures and Tables

**Table 1:** Hydrogen-bond geometry (Å, °)

*D*—H⋯*A*	*D*—H	H⋯*A*	*D*⋯*A*	*D*—H⋯*A*
N1—H1*N*⋯O5^i^	0.91	1.91	2.819 (2)	175
O1—H1*O*⋯O12^ii^	0.84	1.91	2.744 (2)	175
N2—H2*N*⋯O2^iii^	0.91	1.96	2.859 (2)	169
O4—H2*O*⋯O9^iv^	0.84	1.97	2.759 (2)	156
N3—H3*N*⋯O11^v^	0.91	1.95	2.859 (2)	177
O7—H3*O*⋯O6^i^	0.84	1.94	2.757 (2)	166
N4—H4*N*⋯O8^vi^	0.91	1.93	2.843 (2)	176
O10—H4*O*⋯O3^vii^	0.84	1.89	2.722 (2)	169

Symmetry codes: (i) 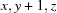; (ii) 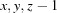; (iii) 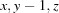; (iv) 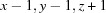; (v) 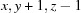; (vi) 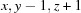; (vii) 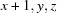.

## supplementary crystallographic information

### S1. Comment

Thiazolidinones are an important group of heterocyclic compounds. Diverse biological activities such as bactericidal, pesticidal, fungicidal, insecticidal, anticonvulsant, tuberculostatic, antiinflammatory, antithyroidal, potentiation of pentobarbital induced sleeping time, *etc*., have been found to be associated with thiazolidinone derivatives (Singh *et al.*, 1981; Bondock *et al.*, 2007; Vicini *et al.*, 2008; Behbehani & Ibrahim, 2012). In this context we report here the synthesis and crystal structure determination of the title compound.

The title compound crystallizes with four independent molecules in the asymmetric unit with similar but significantly different conformations (Fig. 1). This is most distinctly shown by the dihedral angles between the 6- and 5-membererd rings. For molecules 1–4, respectively, these are: 2.6 (1), 1.09 (9), 8.6 (1) and 6.2 (1)°. Intermolecular N—H···O and O—H···O hydrogen bonds (Table 1 and Fig. 2) form sheets running approximately parallel to (101) (Fig. 3).

### S2. Experimental

The title compound was obtained as a major product from a three component reaction of 2-hydroxy-benzaldehyde (1 mmol, 122 mg), thiazolidine-2,4-dione (1 mmol, 117 mg) and 1-aminopropan-2-ol (1 mmol, 75 mg) under reflux. The reaction was monitored by TLC till completion. On cooling the solid product was collected by filteration, dried under vacuum and recrystallized from ethanol to afford yellow crystals in a sufficient quality for X-ray diffraction. *M*.p. 558 K.

### S3. Refinement

H-atoms attached to carbon were placed in calculated positions (C—H = 0.95 Å) while those attached to nitrogen and oxygen were placed in locations derived from a difference map and their coordinates adjusted to give N—H = 0.91 Å and O—H = 0.84 Å. All were included as riding contributions with isotropic displacement parameters 1.2 times those of the attached atoms. Analysis of 852 reflections having I/σ(I) > 13 and chosen from the full data set with *CELL_NOW* showed the crystal to belong to the triclinic system and to consist of one major component (ca. 88%) and two minor components. The raw data were processed using the multi-component version of *SAINT* under control of the two-component orientation file generated by *CELL_NOW*. Reflections from the major twin domain were used for the refinement.

### Crystal data

**Table:**

C_10_H_7_NO_3_S	*Z* = 8
*M**_r_* = 221.23	*F*(000) = 912
Triclinic, *P*1	*D*_x_ = 1.586 Mg m^−^^3^
*a* = 7.2040 (7) Å	Mo *K*α radiation, λ = 0.71073 Å
*b* = 13.6544 (14) Å	Cell parameters from 6790 reflections
*c* = 18.9346 (18) Å	θ = 2.6–28.4°
α = 90.226 (2)°	µ = 0.33 mm^−^^1^
β = 95.531 (2)°	*T* = 150 K
γ = 91.330 (2)°	Parallelepiped, yellow
*V* = 1853.3 (3) Å^3^	0.22 × 0.11 × 0.08 mm

### Data collection

**Table:**

Bruker SMART APEX CCD diffractometer	9869 independent reflections
Radiation source: fine-focus sealed tube	5466 reflections with *I* > 2σ(*I*)
Graphite monochromator	*R*_int_ = 0.074
Detector resolution: 8.3333 pixels mm^-1^	θ_max_ = 29.4°, θ_min_ = 1.8°
φ and ω scans	*h* = −9→9
Absorption correction: multi-scan (*TWINABS*; Sheldrick, 2009)	*k* = −18→18
*T*_min_ = 0.93, *T*_max_ = 0.97	*l* = −26→26
64240 measured reflections	

### Refinement

**Table:**

Refinement on *F*^2^	Primary atom site location: structure-invariant direct methods
Least-squares matrix: full	Secondary atom site location: difference Fourier map
*R*[*F*^2^ > 2σ(*F*^2^)] = 0.051	Hydrogen site location: mixed
*wR*(*F*^2^) = 0.125	H-atom parameters constrained
*S* = 0.93	*w* = 1/[σ^2^(*F*_o_^2^) + (0.0665*P*)^2^] where *P* = (*F*_o_^2^ + 2*F*_c_^2^)/3
9869 reflections	(Δ/σ)_max_ < 0.001
541 parameters	Δρ_max_ = 0.53 e Å^−^^3^
0 restraints	Δρ_min_ = −0.38 e Å^−^^3^

### Special details

**Table:**

Experimental. The diffraction data were obtained from 3 sets of 400 frames, each of width 0.5° in ω, colllected at φ = 0.00, 90.00 and 180.00° and 2 sets of 800 frames, each of width 0.45° in φ, collected at ω = -30.00 and 210.00°. The scan time was 20 sec/frame. Analysis of 852 reflections having I/σ(I) > 13 and chosen from the full data set with *CELL_NOW* (Sheldrick, 2008) showed the crystal to belong to the triclinic system and to consist of one major and two minor components. The second of the minor components was considered to be small enough compared to the others that it could be neglected in the integration. The raw data were processed using the multi-component version of *SAINT* under control of the two-component orientation file generated by *CELL_NOW*.
Geometry. All e.s.d.'s (except the e.s.d. in the dihedral angle between two l.s. planes) are estimated using the full covariance matrix. The cell e.s.d.'s are taken into account individually in the estimation of e.s.d.'s in distances, angles and torsion angles; correlations between e.s.d.'s in cell parameters are only used when they are defined by crystal symmetry. An approximate (isotropic) treatment of cell e.s.d.'s is used for estimating e.s.d.'s involving l.s. planes.
Refinement. Refinement of *F*^2^ against ALL reflections. The weighted *R*-factor *wR* and goodness of fit *S* are based on *F*^2^, conventional *R*-factors *R* are based on *F*, with *F* set to zero for negative *F*^2^. The threshold expression of *F*^2^ > σ(*F*^2^) is used only for calculating *R*-factors(gt) *etc*. and is not relevant to the choice of reflections for refinement. *R*-factors based on *F*^2^ are statistically about twice as large as those based on *F*, and *R*- factors based on ALL data will be even larger. H-atoms attached to carbon were placed in calculated positions (C—H = 0.95 Å) while those attached to nitrogen and oxygen were placed in locations derived from a difference map and their coordinates adjusted to give N—H = 0.91%A and O—H = 0.84%A. All were included as riding contributions with isotropic displacement parameters 1.2 times those of the attached atoms. Trial refinements after all atoms were included indicated that the single component reflection file extracted from the twinned data set gave superior results.

### Fractional atomic coordinates and isotropic or equivalent isotropic displacement parameters (Å^2^)

**Table:**

	*x*	*y*	*z*	*U*_iso_*/*U*_eq_	
S1	0.40644 (8)	0.38610 (4)	0.38644 (3)	0.02299 (14)	
O1	0.6291 (2)	0.42858 (11)	0.12068 (9)	0.0370 (5)	
H1O	0.6690	0.4266	0.0804	0.044*	
O2	0.4643 (2)	0.63917 (11)	0.30031 (8)	0.0254 (4)	
O3	0.2764 (2)	0.47843 (12)	0.49334 (8)	0.0294 (4)	
N1	0.3645 (2)	0.57249 (13)	0.40148 (9)	0.0219 (4)	
H1N	0.3543	0.6318	0.4226	0.026*	
C1	0.5409 (3)	0.34462 (16)	0.22111 (12)	0.0208 (5)	
C2	0.5130 (3)	0.25416 (16)	0.25447 (12)	0.0254 (5)	
H2	0.4697	0.2536	0.3003	0.031*	
C3	0.5467 (3)	0.16666 (17)	0.22248 (13)	0.0294 (6)	
H3	0.5272	0.1066	0.2462	0.035*	
C4	0.6098 (3)	0.16625 (17)	0.15511 (12)	0.0279 (5)	
H4	0.6335	0.1059	0.1329	0.033*	
C5	0.6375 (3)	0.25316 (17)	0.12096 (12)	0.0271 (5)	
H5	0.6813	0.2527	0.0752	0.032*	
C6	0.6020 (3)	0.34172 (16)	0.15285 (12)	0.0241 (5)	
C7	0.5131 (3)	0.43940 (16)	0.25299 (11)	0.0217 (5)	
H7	0.5366	0.4937	0.2237	0.026*	
C8	0.4605 (3)	0.46345 (15)	0.31612 (11)	0.0184 (5)	
C9	0.4339 (3)	0.56730 (16)	0.33623 (11)	0.0195 (5)	
C10	0.3375 (3)	0.48608 (17)	0.43577 (12)	0.0225 (5)	
S2	0.32807 (8)	0.01122 (4)	0.37273 (3)	0.02336 (14)	
O4	0.1704 (2)	−0.04010 (11)	0.64706 (8)	0.0285 (4)	
H2O	0.1434	−0.0225	0.6873	0.034*	
O5	0.3142 (2)	−0.24179 (11)	0.46129 (8)	0.0268 (4)	
O6	0.4279 (2)	−0.07906 (12)	0.25874 (8)	0.0293 (4)	
N2	0.3748 (2)	−0.17408 (13)	0.35499 (9)	0.0218 (4)	
H2N	0.3958	−0.2309	0.3322	0.026*	
C11	0.2175 (3)	0.04760 (16)	0.54267 (11)	0.0192 (5)	
C12	0.1743 (3)	0.04827 (16)	0.61370 (12)	0.0202 (5)	
C13	0.1370 (3)	0.13555 (16)	0.64762 (12)	0.0242 (5)	
H13	0.1067	0.1347	0.6953	0.029*	
C14	0.1441 (3)	0.22250 (17)	0.61207 (13)	0.0267 (5)	
H14	0.1205	0.2818	0.6356	0.032*	
C15	0.1853 (3)	0.22489 (17)	0.54197 (13)	0.0292 (6)	
H15	0.1884	0.2854	0.5174	0.035*	
C16	0.2219 (3)	0.13864 (16)	0.50825 (12)	0.0243 (5)	
H16	0.2509	0.1408	0.4604	0.029*	
C17	0.2538 (3)	−0.04522 (16)	0.50986 (11)	0.0190 (5)	
H17	0.2452	−0.1002	0.5400	0.023*	
C18	0.2977 (3)	−0.06743 (15)	0.44423 (11)	0.0188 (5)	
C19	0.3286 (3)	−0.16940 (16)	0.42378 (11)	0.0202 (5)	
C20	0.3851 (3)	−0.08749 (16)	0.31868 (12)	0.0217 (5)	
S3	0.90516 (8)	0.88184 (4)	−0.11410 (3)	0.02609 (15)	
O7	0.6217 (2)	0.92781 (11)	0.14016 (8)	0.0326 (4)	
H3O	0.5773	0.9192	0.1791	0.039*	
O8	0.7715 (2)	1.13295 (11)	−0.04415 (8)	0.0266 (4)	
O9	1.0061 (2)	0.97420 (12)	−0.22769 (8)	0.0329 (4)	
N3	0.8938 (2)	1.06761 (13)	−0.14051 (9)	0.0222 (4)	
H3N	0.9001	1.1261	−0.1631	0.027*	
C21	0.7074 (3)	0.84107 (16)	0.04052 (11)	0.0199 (5)	
C22	0.6480 (3)	0.83940 (16)	0.10950 (12)	0.0239 (5)	
C23	0.6200 (3)	0.75171 (17)	0.14383 (12)	0.0262 (5)	
H23	0.5818	0.7518	0.1905	0.031*	
C24	0.6476 (3)	0.66414 (17)	0.11018 (13)	0.0292 (6)	
H24	0.6277	0.6042	0.1339	0.035*	
C25	0.7037 (3)	0.66242 (17)	0.04226 (13)	0.0302 (6)	
H25	0.7211	0.6019	0.0191	0.036*	
C26	0.7342 (3)	0.75025 (17)	0.00866 (12)	0.0268 (5)	
H26	0.7748	0.7490	−0.0376	0.032*	
C27	0.7336 (3)	0.93551 (16)	0.00744 (11)	0.0200 (5)	
H27	0.6925	0.9899	0.0327	0.024*	
C28	0.8069 (3)	0.95820 (16)	−0.05322 (11)	0.0202 (5)	
C29	0.8192 (3)	1.06154 (16)	−0.07655 (11)	0.0196 (5)	
C30	0.9443 (3)	0.98161 (17)	−0.17070 (12)	0.0238 (5)	
S4	0.87243 (9)	0.50738 (4)	0.87921 (3)	0.02846 (16)	
O10	1.1108 (2)	0.46387 (11)	0.61625 (8)	0.0274 (4)	
H4O	1.1551	0.4753	0.5776	0.033*	
O11	0.9134 (2)	0.25436 (11)	0.79255 (8)	0.0286 (4)	
O12	0.7722 (3)	0.41381 (13)	0.99199 (9)	0.0386 (5)	
N4	0.8343 (2)	0.32045 (13)	0.89658 (9)	0.0237 (4)	
H4N	0.8161	0.2615	0.9174	0.028*	
C31	1.0119 (3)	0.54867 (16)	0.71490 (11)	0.0197 (5)	
C32	1.0756 (3)	0.55082 (15)	0.64669 (11)	0.0197 (5)	
C33	1.0984 (3)	0.63921 (16)	0.61191 (12)	0.0233 (5)	
H33	1.1400	0.6396	0.5658	0.028*	
C34	1.0609 (3)	0.72617 (17)	0.64428 (12)	0.0269 (5)	
H34	1.0772	0.7864	0.6205	0.032*	
C35	0.9989 (3)	0.72613 (16)	0.71194 (12)	0.0249 (5)	
H35	0.9735	0.7862	0.7342	0.030*	
C36	0.9750 (3)	0.63891 (16)	0.74614 (12)	0.0220 (5)	
H36	0.9323	0.6395	0.7921	0.026*	
C37	0.9820 (3)	0.45351 (16)	0.74680 (11)	0.0203 (5)	
H37	1.0082	0.3991	0.7182	0.024*	
C38	0.9236 (3)	0.42983 (16)	0.80996 (11)	0.0204 (5)	
C39	0.8931 (3)	0.32598 (17)	0.82932 (12)	0.0223 (5)	
C40	0.8174 (3)	0.40666 (18)	0.93258 (12)	0.0259 (5)	

### Atomic displacement parameters (Å^2^)

**Table:**

	*U*^11^	*U*^22^	*U*^33^	*U*^12^	*U*^13^	*U*^23^
S1	0.0304 (3)	0.0206 (3)	0.0193 (3)	0.0001 (2)	0.0096 (2)	0.0009 (2)
O1	0.0658 (13)	0.0232 (9)	0.0265 (10)	0.0031 (8)	0.0273 (9)	0.0037 (7)
O2	0.0356 (10)	0.0195 (8)	0.0230 (9)	0.0006 (7)	0.0114 (7)	0.0011 (7)
O3	0.0359 (10)	0.0323 (9)	0.0221 (9)	−0.0022 (8)	0.0137 (7)	−0.0027 (7)
N1	0.0288 (11)	0.0153 (10)	0.0228 (11)	0.0019 (8)	0.0095 (8)	−0.0044 (8)
C1	0.0215 (12)	0.0188 (12)	0.0230 (12)	0.0014 (9)	0.0065 (9)	−0.0009 (9)
C2	0.0302 (13)	0.0224 (12)	0.0251 (13)	0.0008 (10)	0.0094 (10)	0.0022 (10)
C3	0.0354 (14)	0.0212 (13)	0.0328 (14)	−0.0007 (10)	0.0090 (11)	0.0012 (11)
C4	0.0333 (14)	0.0229 (13)	0.0280 (14)	0.0030 (10)	0.0059 (10)	−0.0052 (10)
C5	0.0323 (14)	0.0264 (13)	0.0239 (13)	0.0019 (10)	0.0099 (10)	−0.0024 (10)
C6	0.0272 (13)	0.0214 (12)	0.0251 (13)	−0.0001 (10)	0.0097 (10)	−0.0003 (10)
C7	0.0251 (12)	0.0188 (12)	0.0221 (12)	−0.0001 (9)	0.0060 (9)	0.0048 (9)
C8	0.0172 (11)	0.0182 (11)	0.0204 (12)	0.0005 (9)	0.0040 (9)	0.0016 (9)
C9	0.0191 (12)	0.0205 (12)	0.0200 (12)	0.0013 (9)	0.0068 (9)	−0.0030 (9)
C10	0.0210 (12)	0.0275 (13)	0.0197 (12)	0.0017 (10)	0.0052 (9)	−0.0020 (10)
S2	0.0310 (3)	0.0213 (3)	0.0192 (3)	0.0022 (2)	0.0094 (2)	0.0018 (2)
O4	0.0447 (10)	0.0212 (9)	0.0225 (9)	0.0020 (7)	0.0180 (7)	−0.0001 (7)
O5	0.0372 (10)	0.0206 (9)	0.0246 (9)	0.0006 (7)	0.0133 (7)	−0.0011 (7)
O6	0.0388 (10)	0.0313 (10)	0.0198 (9)	0.0010 (8)	0.0126 (7)	−0.0017 (7)
N2	0.0296 (11)	0.0193 (10)	0.0174 (10)	0.0010 (8)	0.0071 (8)	−0.0032 (8)
C11	0.0186 (11)	0.0213 (12)	0.0186 (11)	0.0004 (9)	0.0056 (9)	−0.0012 (9)
C12	0.0207 (12)	0.0178 (11)	0.0227 (12)	0.0000 (9)	0.0050 (9)	0.0000 (9)
C13	0.0250 (13)	0.0269 (13)	0.0219 (12)	0.0009 (10)	0.0088 (9)	−0.0064 (10)
C14	0.0305 (14)	0.0181 (12)	0.0330 (14)	0.0026 (10)	0.0102 (10)	−0.0070 (11)
C15	0.0348 (14)	0.0221 (13)	0.0321 (14)	0.0038 (11)	0.0102 (11)	0.0020 (11)
C16	0.0291 (13)	0.0221 (12)	0.0231 (13)	0.0033 (10)	0.0092 (10)	0.0013 (10)
C17	0.0198 (11)	0.0191 (11)	0.0188 (11)	−0.0003 (9)	0.0051 (9)	0.0012 (9)
C18	0.0185 (11)	0.0189 (11)	0.0199 (12)	0.0003 (9)	0.0064 (9)	0.0011 (9)
C19	0.0184 (11)	0.0213 (12)	0.0217 (12)	−0.0002 (9)	0.0055 (9)	−0.0004 (10)
C20	0.0218 (12)	0.0227 (12)	0.0212 (12)	0.0007 (9)	0.0052 (9)	0.0004 (10)
S3	0.0365 (4)	0.0219 (3)	0.0220 (3)	0.0032 (3)	0.0137 (3)	−0.0002 (2)
O7	0.0552 (12)	0.0220 (9)	0.0238 (9)	−0.0029 (8)	0.0209 (8)	−0.0031 (7)
O8	0.0334 (9)	0.0227 (9)	0.0255 (9)	0.0038 (7)	0.0118 (7)	0.0005 (7)
O9	0.0461 (11)	0.0348 (10)	0.0206 (9)	0.0025 (8)	0.0174 (8)	0.0000 (8)
N3	0.0301 (11)	0.0182 (10)	0.0197 (10)	0.0015 (8)	0.0088 (8)	0.0029 (8)
C21	0.0199 (11)	0.0207 (12)	0.0200 (12)	−0.0004 (9)	0.0060 (9)	0.0015 (9)
C22	0.0271 (13)	0.0201 (12)	0.0252 (13)	−0.0013 (10)	0.0076 (10)	−0.0019 (10)
C23	0.0316 (14)	0.0275 (13)	0.0208 (12)	−0.0018 (10)	0.0095 (10)	0.0044 (10)
C24	0.0314 (14)	0.0213 (13)	0.0360 (15)	−0.0019 (10)	0.0093 (11)	0.0085 (11)
C25	0.0369 (15)	0.0181 (12)	0.0374 (15)	0.0009 (10)	0.0129 (11)	0.0002 (11)
C26	0.0317 (14)	0.0252 (13)	0.0249 (13)	0.0012 (10)	0.0100 (10)	0.0001 (10)
C27	0.0234 (12)	0.0175 (11)	0.0199 (12)	−0.0009 (9)	0.0063 (9)	−0.0010 (9)
C28	0.0193 (12)	0.0237 (12)	0.0181 (12)	0.0017 (9)	0.0045 (9)	−0.0001 (9)
C29	0.0177 (11)	0.0223 (12)	0.0195 (12)	0.0009 (9)	0.0044 (9)	0.0015 (10)
C30	0.0275 (13)	0.0257 (13)	0.0192 (12)	−0.0007 (10)	0.0069 (9)	0.0016 (10)
S4	0.0442 (4)	0.0226 (3)	0.0206 (3)	−0.0011 (3)	0.0146 (3)	−0.0020 (2)
O10	0.0387 (10)	0.0227 (9)	0.0236 (9)	0.0050 (7)	0.0162 (7)	0.0006 (7)
O11	0.0428 (10)	0.0209 (9)	0.0240 (9)	0.0017 (7)	0.0135 (7)	0.0009 (7)
O12	0.0602 (13)	0.0379 (11)	0.0212 (9)	−0.0016 (9)	0.0220 (8)	−0.0018 (8)
N4	0.0326 (11)	0.0188 (10)	0.0208 (10)	−0.0032 (8)	0.0087 (8)	0.0022 (8)
C31	0.0193 (11)	0.0188 (11)	0.0215 (12)	0.0001 (9)	0.0040 (9)	0.0011 (9)
C32	0.0219 (12)	0.0182 (11)	0.0199 (12)	0.0021 (9)	0.0066 (9)	−0.0004 (9)
C33	0.0233 (12)	0.0267 (13)	0.0209 (12)	−0.0009 (10)	0.0080 (9)	0.0017 (10)
C34	0.0277 (13)	0.0223 (12)	0.0311 (14)	−0.0022 (10)	0.0050 (10)	0.0068 (11)
C35	0.0286 (13)	0.0171 (12)	0.0295 (13)	−0.0005 (10)	0.0059 (10)	−0.0038 (10)
C36	0.0260 (13)	0.0199 (12)	0.0209 (12)	0.0007 (9)	0.0062 (9)	−0.0021 (9)
C37	0.0238 (12)	0.0179 (11)	0.0201 (12)	0.0023 (9)	0.0059 (9)	−0.0014 (9)
C38	0.0220 (12)	0.0209 (12)	0.0188 (12)	−0.0001 (9)	0.0055 (9)	−0.0001 (9)
C39	0.0235 (12)	0.0229 (12)	0.0211 (12)	−0.0007 (9)	0.0048 (9)	0.0022 (10)
C40	0.0304 (13)	0.0304 (14)	0.0178 (12)	−0.0023 (10)	0.0083 (10)	−0.0017 (10)

### Geometric parameters (Å, º)

**Table:**

S1—C10	1.759 (2)	S3—C28	1.760 (2)
S1—C8	1.770 (2)	S3—C30	1.771 (2)
O1—C6	1.354 (3)	O7—C22	1.362 (3)
O1—H1O	0.8404	O7—H3O	0.8400
O2—C9	1.224 (2)	O8—C29	1.223 (3)
O3—C10	1.218 (2)	O9—C30	1.211 (2)
N1—C10	1.368 (3)	N3—C29	1.373 (3)
N1—C9	1.379 (3)	N3—C30	1.376 (3)
N1—H1N	0.9104	N3—H3N	0.9104
C1—C6	1.406 (3)	C21—C26	1.402 (3)
C1—C2	1.409 (3)	C21—C22	1.413 (3)
C1—C7	1.452 (3)	C21—C27	1.452 (3)
C2—C3	1.375 (3)	C22—C23	1.385 (3)
C2—H2	0.9500	C23—C24	1.380 (3)
C3—C4	1.395 (3)	C23—H23	0.9500
C3—H3	0.9500	C24—C25	1.385 (3)
C4—C5	1.373 (3)	C24—H24	0.9500
C4—H4	0.9500	C25—C26	1.383 (3)
C5—C6	1.389 (3)	C25—H25	0.9500
C5—H5	0.9500	C26—H26	0.9500
C7—C8	1.331 (3)	C27—C28	1.344 (3)
C7—H7	0.9500	C27—H27	0.9500
C8—C9	1.488 (3)	C28—C29	1.483 (3)
S2—C18	1.758 (2)	S4—C38	1.754 (2)
S2—C20	1.769 (2)	S4—C40	1.770 (2)
O4—C12	1.365 (2)	O10—C32	1.357 (2)
O4—H2O	0.8403	O10—H4O	0.8399
O5—C19	1.228 (2)	O11—C39	1.218 (3)
O6—C20	1.210 (2)	O12—C40	1.205 (3)
N2—C20	1.374 (3)	N4—C40	1.372 (3)
N2—C19	1.377 (3)	N4—C39	1.382 (3)
N2—H2N	0.9093	N4—H4N	0.9104
C11—C16	1.407 (3)	C31—C36	1.407 (3)
C11—C12	1.409 (3)	C31—C32	1.412 (3)
C11—C17	1.450 (3)	C31—C37	1.455 (3)
C12—C13	1.395 (3)	C32—C33	1.391 (3)
C13—C14	1.369 (3)	C33—C34	1.378 (3)
C13—H13	0.9500	C33—H33	0.9500
C14—C15	1.388 (3)	C34—C35	1.397 (3)
C14—H14	0.9500	C34—H34	0.9500
C15—C16	1.380 (3)	C35—C36	1.373 (3)
C15—H15	0.9500	C35—H35	0.9500
C16—H16	0.9500	C36—H36	0.9500
C17—C18	1.346 (3)	C37—C38	1.344 (3)
C17—H17	0.9500	C37—H37	0.9500
C18—C19	1.471 (3)	C38—C39	1.483 (3)
			
C10—S1—C8	91.68 (10)	C28—S3—C30	91.93 (10)
C6—O1—H1O	116.9	C22—O7—H3O	109.6
C10—N1—C9	117.22 (19)	C29—N3—C30	117.45 (19)
C10—N1—H1N	122.4	C29—N3—H3N	120.3
C9—N1—H1N	119.8	C30—N3—H3N	122.1
C6—C1—C2	117.1 (2)	C26—C21—C22	116.9 (2)
C6—C1—C7	118.6 (2)	C26—C21—C27	124.8 (2)
C2—C1—C7	124.3 (2)	C22—C21—C27	118.3 (2)
C3—C2—C1	121.8 (2)	O7—C22—C23	122.3 (2)
C3—C2—H2	119.1	O7—C22—C21	116.7 (2)
C1—C2—H2	119.1	C23—C22—C21	121.0 (2)
C2—C3—C4	119.8 (2)	C24—C23—C22	119.9 (2)
C2—C3—H3	120.1	C24—C23—H23	120.1
C4—C3—H3	120.1	C22—C23—H23	120.1
C5—C4—C3	119.9 (2)	C23—C24—C25	120.9 (2)
C5—C4—H4	120.0	C23—C24—H24	119.5
C3—C4—H4	120.0	C25—C24—H24	119.5
C4—C5—C6	120.5 (2)	C26—C25—C24	118.9 (2)
C4—C5—H5	119.8	C26—C25—H25	120.5
C6—C5—H5	119.8	C24—C25—H25	120.5
O1—C6—C5	121.8 (2)	C25—C26—C21	122.3 (2)
O1—C6—C1	117.2 (2)	C25—C26—H26	118.9
C5—C6—C1	121.0 (2)	C21—C26—H26	118.9
C8—C7—C1	131.2 (2)	C28—C27—C21	130.3 (2)
C8—C7—H7	114.4	C28—C27—H27	114.9
C1—C7—H7	114.4	C21—C27—H27	114.9
C7—C8—C9	121.54 (19)	C27—C28—C29	120.5 (2)
C7—C8—S1	129.03 (17)	C27—C28—S3	129.85 (18)
C9—C8—S1	109.41 (15)	C29—C28—S3	109.61 (15)
O2—C9—N1	123.5 (2)	O8—C29—N3	123.4 (2)
O2—C9—C8	126.0 (2)	O8—C29—C28	125.8 (2)
N1—C9—C8	110.46 (18)	N3—C29—C28	110.74 (19)
O3—C10—N1	125.1 (2)	O9—C30—N3	125.5 (2)
O3—C10—S1	123.85 (18)	O9—C30—S3	124.39 (19)
N1—C10—S1	111.07 (16)	N3—C30—S3	110.10 (15)
C18—S2—C20	91.79 (10)	C38—S4—C40	91.88 (11)
C12—O4—H2O	100.4	C32—O10—H4O	108.3
C20—N2—C19	117.47 (19)	C40—N4—C39	117.62 (19)
C20—N2—H2N	118.7	C40—N4—H4N	121.3
C19—N2—H2N	123.8	C39—N4—H4N	121.0
C16—C11—C12	116.9 (2)	C36—C31—C32	117.46 (19)
C16—C11—C17	124.3 (2)	C36—C31—C37	124.5 (2)
C12—C11—C17	118.78 (19)	C32—C31—C37	118.0 (2)
O4—C12—C13	122.1 (2)	O10—C32—C33	121.60 (19)
O4—C12—C11	116.80 (19)	O10—C32—C31	117.65 (19)
C13—C12—C11	121.1 (2)	C33—C32—C31	120.7 (2)
C14—C13—C12	120.0 (2)	C34—C33—C32	120.1 (2)
C14—C13—H13	120.0	C34—C33—H33	120.0
C12—C13—H13	120.0	C32—C33—H33	120.0
C13—C14—C15	120.7 (2)	C33—C34—C35	120.3 (2)
C13—C14—H14	119.6	C33—C34—H34	119.8
C15—C14—H14	119.6	C35—C34—H34	119.8
C16—C15—C14	119.4 (2)	C36—C35—C34	119.7 (2)
C16—C15—H15	120.3	C36—C35—H35	120.1
C14—C15—H15	120.3	C34—C35—H35	120.1
C15—C16—C11	121.9 (2)	C35—C36—C31	121.7 (2)
C15—C16—H16	119.0	C35—C36—H36	119.2
C11—C16—H16	119.0	C31—C36—H36	119.2
C18—C17—C11	131.5 (2)	C38—C37—C31	130.7 (2)
C18—C17—H17	114.2	C38—C37—H37	114.7
C11—C17—H17	114.2	C31—C37—H37	114.7
C17—C18—C19	120.88 (19)	C37—C38—C39	120.8 (2)
C17—C18—S2	129.02 (17)	C37—C38—S4	128.92 (18)
C19—C18—S2	110.10 (15)	C39—C38—S4	110.26 (15)
O5—C19—N2	123.4 (2)	O11—C39—N4	123.4 (2)
O5—C19—C18	126.0 (2)	O11—C39—C38	126.6 (2)
N2—C19—C18	110.57 (19)	N4—C39—C38	110.0 (2)
O6—C20—N2	125.5 (2)	O12—C40—N4	125.5 (2)
O6—C20—S2	124.48 (18)	O12—C40—S4	124.3 (2)
N2—C20—S2	110.04 (16)	N4—C40—S4	110.20 (16)
			
C6—C1—C2—C3	1.1 (3)	C26—C21—C22—O7	−179.87 (19)
C7—C1—C2—C3	−178.1 (2)	C27—C21—C22—O7	−0.7 (3)
C1—C2—C3—C4	−0.2 (4)	C26—C21—C22—C23	0.7 (3)
C2—C3—C4—C5	−0.1 (4)	C27—C21—C22—C23	179.8 (2)
C3—C4—C5—C6	−0.4 (4)	O7—C22—C23—C24	179.6 (2)
C4—C5—C6—O1	179.6 (2)	C21—C22—C23—C24	−0.9 (4)
C4—C5—C6—C1	1.3 (4)	C22—C23—C24—C25	0.2 (4)
C2—C1—C6—O1	−179.96 (19)	C23—C24—C25—C26	0.8 (4)
C7—C1—C6—O1	−0.8 (3)	C24—C25—C26—C21	−1.0 (4)
C2—C1—C6—C5	−1.6 (3)	C22—C21—C26—C25	0.3 (3)
C7—C1—C6—C5	177.6 (2)	C27—C21—C26—C25	−178.8 (2)
C6—C1—C7—C8	−178.6 (2)	C26—C21—C27—C28	−8.9 (4)
C2—C1—C7—C8	0.5 (4)	C22—C21—C27—C28	172.0 (2)
C1—C7—C8—C9	−177.5 (2)	C21—C27—C28—C29	180.0 (2)
C1—C7—C8—S1	0.8 (4)	C21—C27—C28—S3	−2.1 (4)
C10—S1—C8—C7	−174.7 (2)	C30—S3—C28—C27	178.1 (2)
C10—S1—C8—C9	3.77 (16)	C30—S3—C28—C29	−3.85 (16)
C10—N1—C9—O2	−180.0 (2)	C30—N3—C29—O8	179.0 (2)
C10—N1—C9—C8	1.5 (3)	C30—N3—C29—C28	−0.6 (3)
C7—C8—C9—O2	−3.5 (3)	C27—C28—C29—O8	2.0 (3)
S1—C8—C9—O2	177.87 (19)	S3—C28—C29—O8	−176.31 (18)
C7—C8—C9—N1	175.0 (2)	C27—C28—C29—N3	−178.43 (19)
S1—C8—C9—N1	−3.7 (2)	S3—C28—C29—N3	3.3 (2)
C9—N1—C10—O3	−179.6 (2)	C29—N3—C30—O9	177.5 (2)
C9—N1—C10—S1	1.4 (3)	C29—N3—C30—S3	−2.3 (2)
C8—S1—C10—O3	177.9 (2)	C28—S3—C30—O9	−176.3 (2)
C8—S1—C10—N1	−3.04 (17)	C28—S3—C30—N3	3.57 (17)
C16—C11—C12—O4	−179.87 (18)	C36—C31—C32—O10	−179.76 (19)
C17—C11—C12—O4	0.2 (3)	C37—C31—C32—O10	−2.1 (3)
C16—C11—C12—C13	0.3 (3)	C36—C31—C32—C33	−0.7 (3)
C17—C11—C12—C13	−179.60 (19)	C37—C31—C32—C33	176.92 (19)
O4—C12—C13—C14	179.5 (2)	O10—C32—C33—C34	179.8 (2)
C11—C12—C13—C14	−0.7 (3)	C31—C32—C33—C34	0.8 (3)
C12—C13—C14—C15	1.0 (4)	C32—C33—C34—C35	−0.3 (3)
C13—C14—C15—C16	−0.9 (4)	C33—C34—C35—C36	−0.2 (3)
C14—C15—C16—C11	0.5 (4)	C34—C35—C36—C31	0.2 (3)
C12—C11—C16—C15	−0.2 (3)	C32—C31—C36—C35	0.2 (3)
C17—C11—C16—C15	179.7 (2)	C37—C31—C36—C35	−177.2 (2)
C16—C11—C17—C18	0.1 (4)	C36—C31—C37—C38	−2.8 (4)
C12—C11—C17—C18	−180.0 (2)	C32—C31—C37—C38	179.8 (2)
C11—C17—C18—C19	179.9 (2)	C31—C37—C38—C39	176.4 (2)
C11—C17—C18—S2	0.2 (4)	C31—C37—C38—S4	−3.4 (4)
C20—S2—C18—C17	178.4 (2)	C40—S4—C38—C37	−179.0 (2)
C20—S2—C18—C19	−1.28 (16)	C40—S4—C38—C39	1.07 (17)
C20—N2—C19—O5	179.6 (2)	C40—N4—C39—O11	179.9 (2)
C20—N2—C19—C18	0.3 (3)	C40—N4—C39—C38	−0.9 (3)
C17—C18—C19—O5	1.9 (3)	C37—C38—C39—O11	−1.0 (4)
S2—C18—C19—O5	−178.41 (18)	S4—C38—C39—O11	178.9 (2)
C17—C18—C19—N2	−178.90 (19)	C37—C38—C39—N4	179.8 (2)
S2—C18—C19—N2	0.8 (2)	S4—C38—C39—N4	−0.3 (2)
C19—N2—C20—O6	178.2 (2)	C39—N4—C40—O12	−178.2 (2)
C19—N2—C20—S2	−1.3 (2)	C39—N4—C40—S4	1.7 (3)
C18—S2—C20—O6	−178.1 (2)	C38—S4—C40—O12	178.3 (2)
C18—S2—C20—N2	1.45 (16)	C38—S4—C40—N4	−1.56 (18)

### Hydrogen-bond geometry (Å, º)

**Table:**

*D*—H···*A*	*D*—H	H···*A*	*D*···*A*	*D*—H···*A*
N1—H1*N*···O5^i^	0.91	1.91	2.819 (2)	175
O1—H1*O*···O12^ii^	0.84	1.91	2.744 (2)	175
N2—H2*N*···O2^iii^	0.91	1.96	2.859 (2)	169
O4—H2*O*···O9^iv^	0.84	1.97	2.759 (2)	156
N3—H3*N*···O11^v^	0.91	1.95	2.859 (2)	177
O7—H3*O*···O6^i^	0.84	1.94	2.757 (2)	166
N4—H4*N*···O8^vi^	0.91	1.93	2.843 (2)	176
O10—H4*O*···O3^vii^	0.84	1.89	2.722 (2)	169

Symmetry codes: (i) *x*, *y*+1, *z*; (ii) *x*, *y*, *z*−1; (iii) *x*, *y*−1, *z*; (iv) *x*−1, *y*−1, *z*+1; (v) *x*, *y*+1, *z*−1; (vi) *x*, *y*−1, *z*+1; (vii) *x*+1, *y*, *z*.
