# Supplementary figures and images for: Crystal structure of (5Z)-5-(2-hy­droxy­benzyl­idene)-1,3-thia­zolidine-2,4-dione
Source: Acta Crystallogr E Crystallogr Commun. 2015 Nov 21;71(Pt 12):o965–6. doi: 10.1107/S2056989015021908 (PMC4719925; doi:10.1107/S2056989015021908)

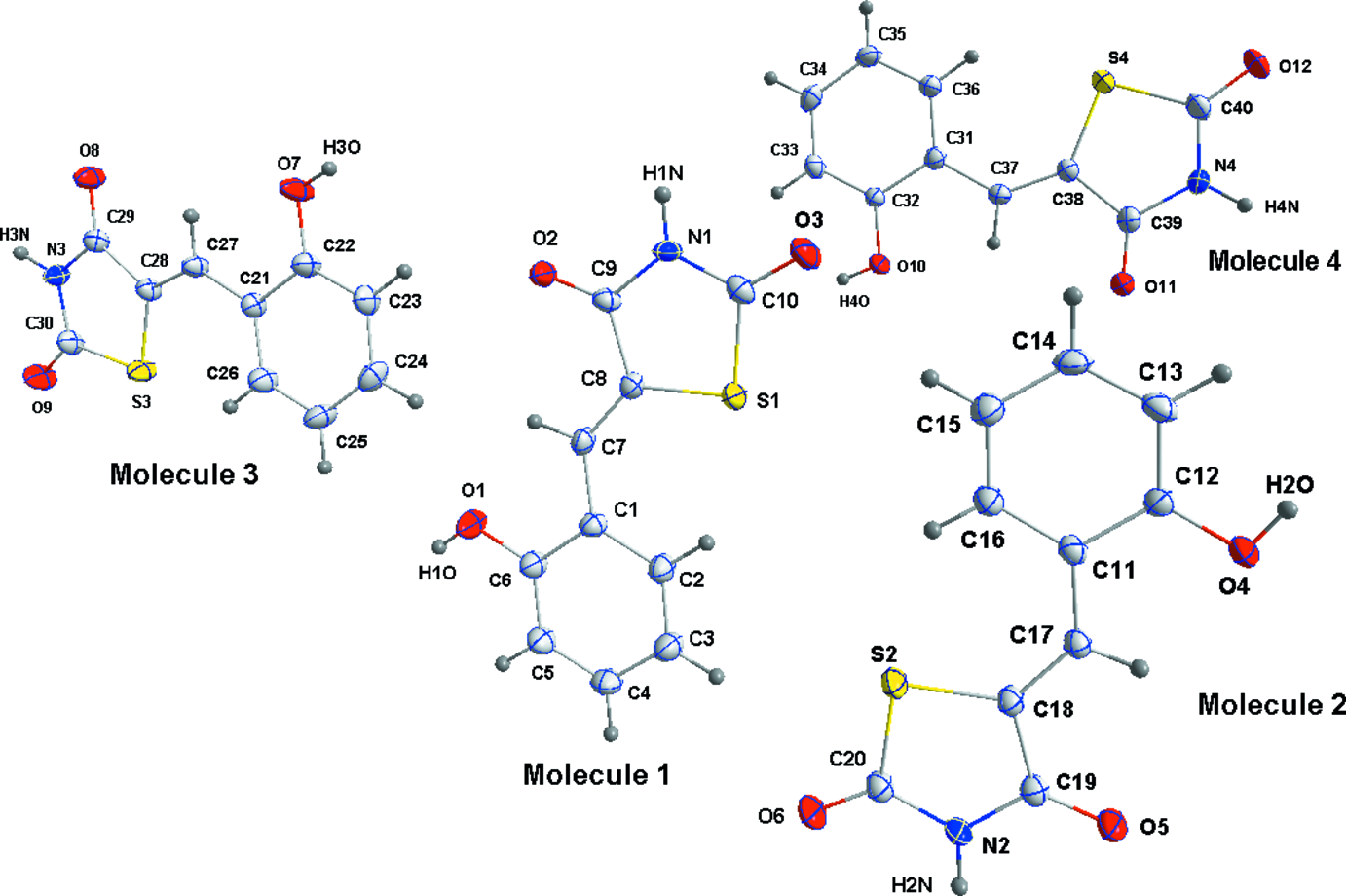

Supplement: Supplementary file 4 [file e-71-0o965-fig1.tif]

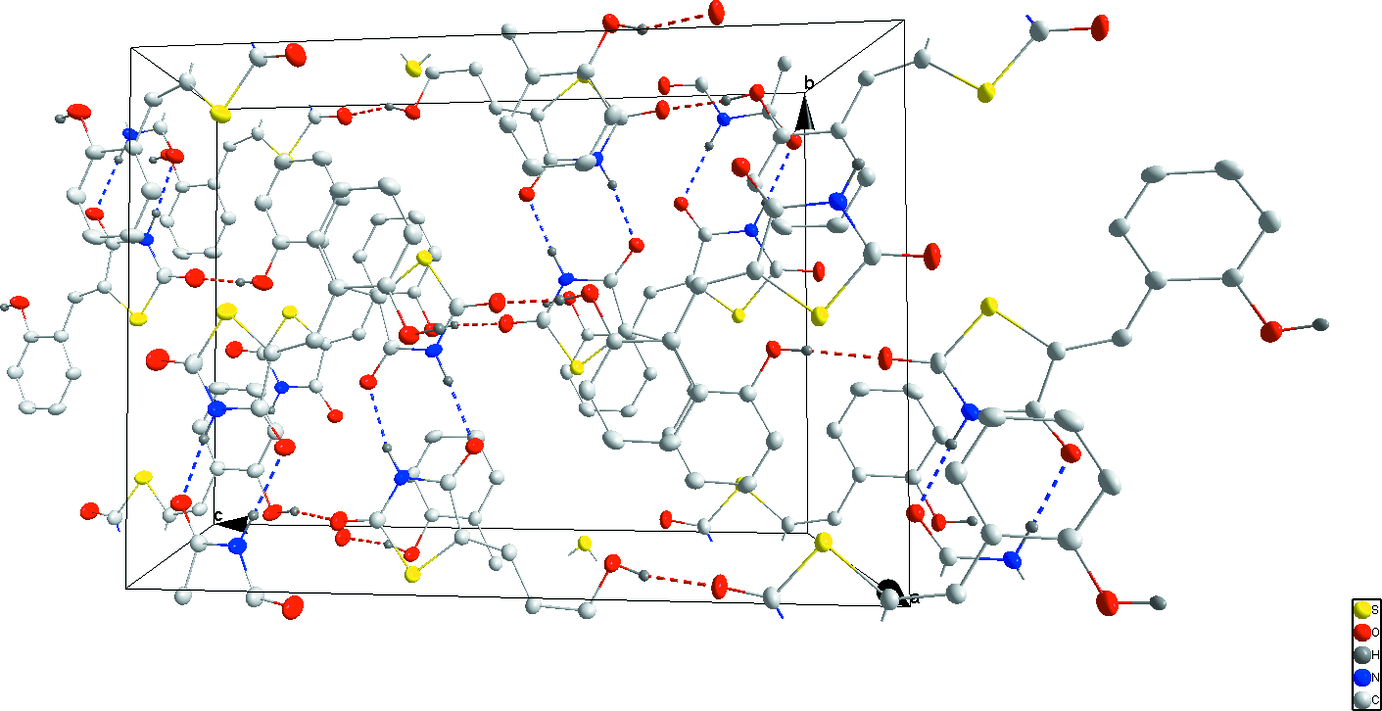

Supplement: Supplementary file 5 [file e-71-0o965-fig2.tif]

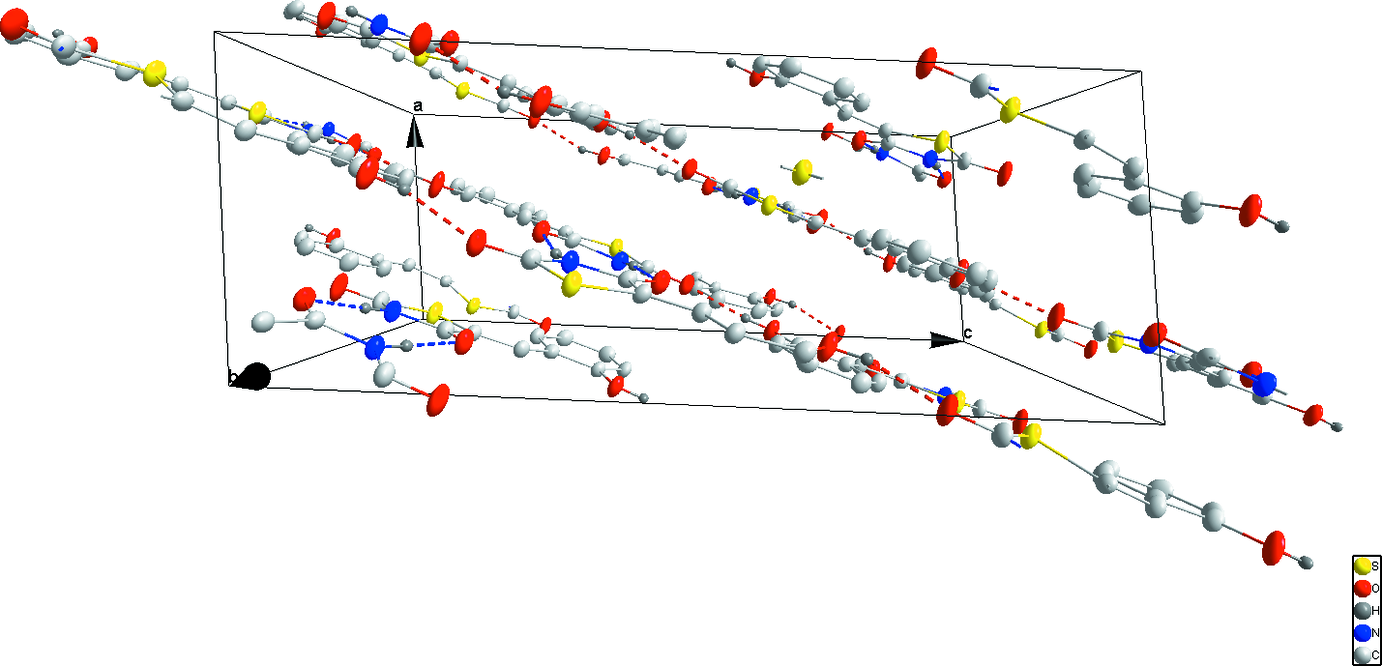

Supplement: Supplementary file 6 [file e-71-0o965-fig3.tif]
